# Reliability and Validity of the Diagnostic Instrument on Adaptive Behaviour: A New Instrument Measuring Adaptive Behaviour in People With Moderate, Severe or Profound Intellectual Disability

**DOI:** 10.1111/jar.70150

**Published:** 2025-11-14

**Authors:** Hinke Elisabet Drijver, Robert Didden, Carlo Schuengel

**Affiliations:** ^1^ Faculty of Behavioural and Movement Sciences, Section of Clinical Child and Family Studies & Amsterdam Public Health Research Institute Vrije Universiteit Amsterdam Amsterdam the Netherlands; ^2^ 's Heeren Loo Amersfoort the Netherlands; ^3^ Behavioural Science Institute Radboud University Nijmegen the Netherlands

**Keywords:** adaptive behaviour, DIAB, moderate intellectual disability, profound intellectual disability, severe intellectual disability

## Abstract

**Background:**

Assessment of adaptive behaviour of people with moderate to profound intellectual disability is hampered by limited variation in scores within this range. We evaluated measurement properties of the Diagnostic Instrument on Adaptive Behaviour (DIAB), which was developed for this population.

**Method:**

The DIAB was completed by two care staff members for 73 adults (age 19–84) grouped by level of intellectual disability (i.e., moderate, severe or profound intellectual disability) along with Dutch normed measures of adaptive and motor functioning and a global rating.

**Results:**

Inter‐rater (ICC = 0.94) and test–retest (ICC = 0.96) reliability met standards. DIAB scores correlated highly with those of the two Dutch instruments (*r* = 0.90; *r* = 0.77). Associations between measures were consistent with convergent and discriminant validity. DIAB scores differed between three severity levels of ID (*p* < 0.001).

**Conclusion:**

The DIAB exhibited promising reliability, convergent, discriminant, concurrent, and known group validity.

**Summary**

- To address the needs of people with intellectual disability it is important to regularly assess their adaptive behaviour by evaluating their daily living skills.
- To assess the severity of intellectual disability and for refined insight in the adaptive skills in people with moderate, severe and profound intellectual disability, we developed the DIAB.
- Two direct care staff members of 73 clients with a profound, severe or moderate intellectual disability completed the new instrument together with other instruments of adaptive behaviour and motor skills.
- Based on promising reliability and validity, the DIAB might be a feasible way to help caregivers get insight into the severity of intellectual disability and to plan and evaluate support for people with moderate, severe or profound intellectual disability.

## 1 Introduction

An intellectual disability is characterised by significant limitations in intellectual functioning and in adaptive behaviour in conceptual, social, and practical skills domains (American Psychiatric Association 2022; Schalock et al. 2021; Tassé and Balboni 2021; Tassé and Kim 2023; World Health Organization/WHO 2022). According to the diagnostic guidelines of the DSM‐5‐TR (American Psychiatric Association 2022) and ICD‐11 (WHO 2022), the assessment of severity levels of intellectual disability and related support needs should focus on adaptive behaviour. In people with moderate and in particular severe or profound intellectual disability, intellectual functioning is difficult to assess due to floor effects and limitations in motor or sensory functions (Maes et al. 2021; WHO 2022). Whilst adaptive behaviour is arguably more relevant than intellectual functioning for identifying support needs in this population (Tassé and Kim 2023), differences in adaptive behaviour between individuals may, however, also be difficult to assess in this population. Instruments for assessing adaptive behaviour need to be able to differentiate adaptive behaviour in this range. This study tested a new instrument, the ‘Diagnostisch Instrument Adaptief Gedrag’ (DIAG) (English: Diagnostic Instrument on Adaptive Behaviour: DIAB), that was specifically developed for supporting severity classification, describing adaptive functioning and support needs, and providing input for support planning based on adaptive behaviour functioning levels.

### 1.1 Limitations of Current Instruments on Adaptive Behaviour

In the Netherlands, there are currently three measures available for assessing adaptive behaviour for adults with intellectual disability. The first and most widely used instrument is a Dutch adaptation of the Cain‐Levine Social Competency Scale (Cain et al. 1963), which is called the ‘Sociale Redzaamheidsschaal‐Z’ (SRZ, English: Social Competency Scale for People with Intellectual Disabilities; Kraijer et al. 2004). This measure is short and easy to administer to direct care staff. The SRZ differentiates well between moderate and severe intellectual disability levels, based on norm groups of people with these severity levels (Egberink and De Leng 2024). The SRZ was not specifically designed for and normed for adults with profound intellectual disability. It has not been updated since the 1970s and may be out of step with the needs of individuals to participate in society today. Moreover, the SRZ norm data are outdated (Egberink and De Leng 2024).

The second instrument is the ABAS‐3 (Adaptive Behaviour Assessment System; Harrison and Oakland 2015, 2020), which was translated in Dutch (Kreemers et al. 2024). The instrument is comprehensive and congruent with the DSM‐5‐TR definition of adaptive behaviour. Dutch general population norms for the ABAS‐3 differentiate in functioning to two standard deviations below the mean (standard score of 59). No differentiation can be made in the moderate to profound range of intellectual disability (Kreemers et al. 2024) due to the absence of people with these severity levels in the norm group (see Harrison and Oakland 2015, 2020).

The third instrument is the Dutch version of the Vineland‐3‐NL (Sparrow et al. 2016, 2020), which is comprehensive and congruent with the DSM‐5‐TR definition of adaptive behaviour. The Vineland‐3 was not specifically designed for people with moderate, severe, and profound intellectual disability and has norm data based on the general United States population, resulting in a very small number (0.9% [*n* = 23] of a total *n* of 2560) of people with an intellectual disability included in the norm groups (Sparrow et al. 2016). Schalock et al. (2021) noted that standardised scores at the low end of the scale of adaptive behaviour measures present challenges with stability and reliability for people with severe and profound intellectual disability, hampering the diagnostic process. Whilst the Vineland manual suggests that age equivalents might offer an alternative, this practise has psychometric objections (Gengoux 2021) and may also misrepresent the disharmonious and non‐linear developmental pathway of people with severe to profound intellectual disability (Visser et al. 2017; Vlaskamp 2005).

### 1.2 Aims and Features of the DIAB

To address the limitations of currently available instruments in the Netherlands developed for the general population, the DIAB was constructed. The goal was to overcome floor effects and offer an instrument that would be brief, self‐explanatory, and easy to administer repeatedly. Generally, people with lower levels of adaptive behaviour need more support (Obremski 2013), usually requiring more intensive care and specialised care staff, than people with higher levels of functioning. Therefore, eligibility for intensive long‐term care is often determined on the basis of an assessment of the severity of intellectual disability, such as in the Netherlands under the long‐term care act (Wet Langdurige Zorg; Woittiez et al. 2018). The WHO (2022) stresses the importance of using appropriately normed, standardised and individually administered instruments of adaptive behaviour to determine severity levels of intellectual disability.

For assessing adaptive behaviour in people with moderate, severe or profound intellectual disability, instruments should not only be current, reliable, and valid (WHO 2022) but also provide insight into the concrete skills and steps towards mastering these, that may be trained or supported. Accordingly, each item in the DIAB addresses one skill, ordered into steps towards ascending levels of mastery of that skill. The DIAB was based on the definition of adaptive behaviour of the American Association on Intellectual and Developmental Disabilities (Schalock et al. 2021), adopted by the DSM‐5‐TR (American Psychiatric Association 2022), as a range of practical, social and conceptual skills to be performed in daily life. It was also specifically developed for people with moderate, severe and profound intellectual disability (for information on the development of the DIAB, see Supplement). It covers three core skill domains: conceptual skills, practical skills and social skills on which the total DIAB scale score is based. The domain ‘motor skills’ was added as a supplemental and optional domain.

### 1.3 The Motor Skill Domain in the DIAB

Although motor skills are not included in the definition of adaptive behaviour, adaptive behaviour and motor skills are associated (Delgado‐Lobete et al. 2021; Fears et al. 2022; McCulloch et al. 2024). Adding an optional motor domain to an instrument for adaptive behaviour is potentially useful in the care for people with moderate to profound intellectual disability. A person with low motor skills and low adaptive behaviour may need physical support and possibly also instructional and behavioural support to complete certain adaptive behaviours. A person with low motor skills and medium to high adaptive behaviour skills, may need physical support and possibly less instructional and behavioural support to complete certain adaptive behaviours. A person with medium to high motor skills and low adaptive behaviour may need instructional and behavioural support only. These situations are bound to occur, given the high prevalence amongst persons with severe to very severe intellectual disability of motor disorders (e.g., cerebral palsy; Nakken and Vlaskamp 2007) and of low motor skills (Jeoung 2018; Katz and Lazcano‐Ponce 2008). Also, at the same time motor skills at more severe levels of intellectual disability may still vary (Nakken and Vlaskamp 2007).

### 1.4 Aims of the Current Study

This study focuses on the measurement properties of the DIAB in a sample of adults with moderate to profound intellectual disability. Such measurement properties need to be evaluated according to benchmarks and criteria that apply specifically to this field (e.g., Bakker et al. 2019). Hence, we evaluated reliability according to the most recent and extensive systematic review on 14 adaptive behaviour measures by Floyd et al. (2015). Floyd et al. based their review on previous reviews on adaptive behaviour measures as well as standards for educational and psychological testing (American Educational Research Association, American Psychological Association, and National Council on Measurement in Education 2014). For convergent validity, a guideline from educational assessment was used (see Odom and Morrow 2006).

First, we assessed known‐group validity by evaluating differences in mean total DIAB scores across three groups with different severity levels (i.e., moderate, severe and profound). Second, convergent validity of the DIAB (three core skill domains: conceptual, practical, and social) was assessed against the SRZ (Kraijer et al. 2004), which is the most often used and familiar instrument for this population in the Netherlands. Also, similar to the DIAB, it is brief. The optional domain motor skills was assessed against the ‘Schaal voor Motoriek’ (SMZ; Translation: Motor Scale for People with Intellectual Disabilities; Kraijer and Kema 1994). Third, concurrent validity was evaluated against global ratings by direct care staff of adaptive behaviour levels of their clients. Fourth, to assess both convergent and discriminant validity, the pattern of associations was evaluated between mean DIAB domain scores and various domain scores of the SRZ and the SMZ measuring similar versus different domains (i.e., subconstructs). We also included staff's global ratings of adaptive behaviour in this analysis. We expected correlations between domains measuring similar skills to be high and correlations between domains measuring different skills to be moderate or weak. For the interpretation of correlation coefficients, we used effect size qualifications by Dancey and Reidy (2007) in accordance with Schober et al. (2018). Fifth, we assessed interrater reliability of the mean total and mean DIAB domain scores. Finally, we evaluated test–retest reliability of the mean total and mean DIAB domain scores.

## 2 Materials and Methods

### 2.1 Setting and Participants

Data were collected between November 2021 and October 2022. The sample consisted of 73 adults with a diagnosis of moderate to profound intellectual disability receiving 24/7 care and support in 11 residential care facilities across the Netherlands. A total of 39 men and 34 women with a mean age of 41 (median 37; range 19 to 84 years; SD = 16.8) participated. A subgroup of 24 participants was classified with moderate, 30 with severe, and 19 with profound intellectual disability. A group of 29 participants lived in the three northern provinces of the Netherlands, seven lived in the two eastern provinces, and 37 lived in the four western provinces (see Table 1). The Netherlands is a rather small country with relatively modest intranational sociocultural differences compared to other, much larger countries in Europe (Kaasa et al. 2014). Moreover, the participants in this study all lived in residential care facilities and received 24 h per day care support, financially based on a national law called the ‘long‐term care act’ (Wet Langdurige Zorg; Woittiez et al. 2018), for these reasons not much socioeconomic differences are expected to be seen between these individuals.

**TABLE 1:** Demographics and scores on DIAB grouped by severity level.

Measure		Moderate intellectual disability	Severe intellectual disability	Profound intellectual disability	Total group^a^
*N*		24	30	19	73
Gender	Men	10	20	9	39 (53%)
	Women	14	10	10	34 (47%)
Age	*M* (SD)	47.71 (17.92)	37.87 (16.79)	37.16 (12.95)	40.92 (16.77)
	Range	19–84	19–75	22–72	19–84
Region	North^b^	13	10	6	29
	East^c^	5	2	0	7
	South^d^	0	0	0	0
	West^e^	6	18	13	37
DIAB mean total item score	*M* (SD)	3.18 (0.53)	2.42 (0.57)	1.51 (0.24)	2.42 (0.81)
	Range	2.06–3.97	1.53–3.81	1.14–1.96	1.14–3.97
DIAB CON	*M* (SD)	3.47 (0.59)	2.70 (0.63)	1.67 (0.28)	2.67 (0.88)
	Range	2.53–4.27	1.67–4.00	1.14–2.27	1.14–4.27
DIAB PRAC	*M* (SD)	3.13 (0.59)	2.26 (0.68)	1.43 (0.28)	2.32 (0.87)
	Range	1.68–4.08	1.48–4.08	1.08–1.96	1.08–4.08
DIAB SOC	*M* (SD)	2.93 (0.58)	2.30 (0.63)	1.44 (0.32)	2.28 (0.79)
	Range	2.00–4.08	1.23–3.70	1.07–2.48	1.07–4.08
DIAB MOT	*M* (SD)	3.74 (0.55)	3.24 (0.80)	2.26 (0.61)	3.14 (0.88)
	Range	2.27–4.64	1.82–4.82	1.18–3.36	1.18–4.82

*Note:* All DIAB measures are based on scores by Informant One (*N* = 70, 3 missing).

Abbreviations: *M* = mean score; *N* = number of participants; SD = standard deviation.

^a^ DIAB scores are based on scores by informant one.

^b^ North: Provinces of Friesland, Groningen, Drenthe.

^c^ East: Provinces of Overijssel and Gelderland.

^d^ South: Provinces of Limburg, Noord Brabant and Zeeland.

^e^ West: Provinces of Flevoland, Utrecht, Noord Holland and Zuid Holland.

Informants, a set of two members of the direct care staff (26 men, 114 women) of each participating client working at least 3 months with the client, filled out questionnaires about their client. All care staff had an associate or bachelor degree in applied science. Generally, half of the informants were the assigned staff member of the client (the member of the care staff team who was responsible for the preparation, implementation, and monitoring of the personal care plans and the primary point of contact for family members); the other half of the informants were other care staff members working with the same client in the same team.

**FIGURE 1:**
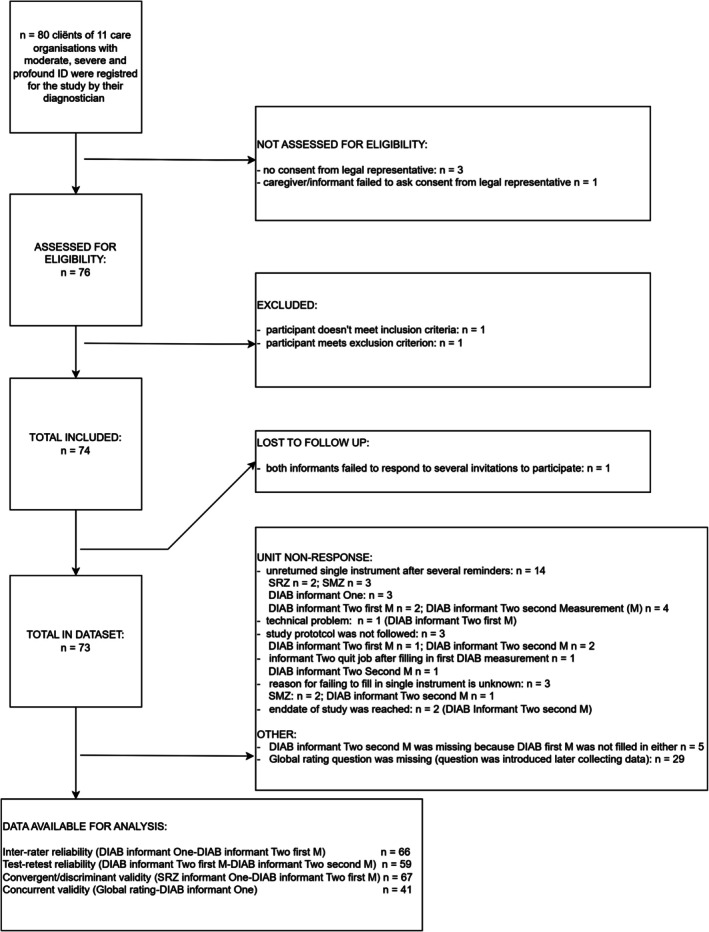
Participant flow.

The consulting diagnostician assessed the inclusion and exclusion criteria.^1^ Inclusion criteria were a diagnosis of moderate, severe, or profound intellectual disability and age of 18 years or older. Participants were functioning stably according to the diagnostician. Exclusion criteria were: (1) severe immobilising short‐term or long‐term condition (severely affecting all motor skills), (2) active psychosis or manic episodes (if suppressed by medication clients could participate), (3) severe visual impairment (functionally blind), (4) severe hearing loss (hearing threshold of > 71 dB), (5) severe and uncontrolled epilepsy or (6) severe motor impairment affecting all four limbs (for example: bilateral paresis severely affecting four limbs; quadriplegia). In these cases, the severe chronic conditions are expected to severely limit adaptive behaviour across all domains, which might inflate estimates of cross‐domain correlations and interrater reliability. The participant flow (Figure 1) shows the inclusion and exclusion of participants.

### 2.2 Procedure

Permission was obtained from the ethical review board (Vaste Commissie Wetenschap en Ethiek/VCWE, VCWE‐2021‐196R1) of the Vrije Universiteit Amsterdam. Participating facilities were members of a collaboration of northern Dutch care facilities for people with disabilities. Facilities from other parts of the Netherlands were approached and recruited for participation by the research team. Participants were selected by the consulting diagnostician of the participant.

Consulting diagnosticians were asked to estimate the severity of intellectual disability. Given the limitations of extant measures of intellectual and adaptive behaviour for people with moderate to profound intellectual disability, estimates of the severity of the intellectual disability were made based on three criteria: (a) recent IQ test or developmental test aimed at children, (b) adaptive behaviour scale results (if available and not older than 5 years), and (c) clinical judgement of the consulting diagnostician. The DSM‐IV bandwidths for IQ and cognitive age equivalents were used for the level of severity (which are listed as moderate: total IQ 35/40–50/55, severe: total IQ 20/25–35/40, profound: total IQ < 20/25; or age equivalents, moderate: 4.0–7.0 years, severe: 2.0–4.0 years, profound: < 2.0 years; American Psychiatric Association 2000; WHO 2022). Estimates of adaptive behaviour functioning were based on norms (if available) for the instrument used or age equivalent scores. Clinical judgement was based on typical behaviour and care intensity per severity level. To support clinical judgement, we provided a short version of a behavioural indicator scheme (based on: American Psychiatric Association 2022 and Tassé et al. 2019). When results were ambiguous, the diagnostician's clinical judgement was the deciding factor.

After selection, inclusion, and classification by the diagnostician, two members of the direct care staff (referred to as: Informant One and Informant Two) of each client completed the DIAB together with an instrument assessing adaptive skills (i.e., SRZ) and an instrument measuring motor skills (i.e., SMZ). A global rating question evaluating the overall adaptive behaviour level as estimated by Informant One was used to assess concurrent validity.

Informant One completed the DIAB, SRZ, and SMZ for convergent and discriminant validity. Informant Two completed the DIAB at two different moments (in the same week as Informant One, and again 3 weeks later) for test–retest reliability. Inter‐rater reliability was assessed by comparing DIAB scores of Informant One and those of Informant Two. Instructions were given in writing as part of the assessment packages. After completing half of the items of the DIAB, the most important instructions for completing this instrument were repeated. The DIAB as well as the global rating question were administered using Qualtrics (XM 2021) by sending a personalised but anonymized link by e‐mail to the informants. The SRZ was taken using a Dutch automated platform for administering psychodiagnostic instruments (i.e., BergOp; Praktikon. 2021) sending a link by e‐mail via the BergOp platform, whereas the SMZ was taken via paper‐and‐pencil which was sent by post.

### 2.3 Instruments

#### 2.3.1 Adaptive Behaviour Assessed With the DIAB

Characteristics of the DIAB to be developed and the content validity of the first versions were investigated by consulting a panel of 19 diagnosticians working with people with intellectual disability, leading to the DIAB used in the current study (also see Supplement). The DIAB contains 67 items in total, with the conceptual domain (CON) containing 22, the practical domain (PRAC) containing 25, and the social domain (SOC) containing 20 items. The motor domain (MOT) is optional and contains 11 items. Hence, the core DIAB consists of the 67 items covering the conceptual, practical, and social domains. Items are represented in a so‐called ‘ordered category format’: 62 items of the 67 items have five scoring options and  five items have four scoring options, differentiating between ascending levels of mastery of an adaptive skill, and resulting in raw scores from 1 to 5 per item (for more information about how items with four options were recalibrated, see Statistical analyses). All 11 items of the motor domain have five scoring options. Each item belongs to one of the four skill domains. Akin to Likert‐type scaling, numeric values for the level of mastery of each skill are averaged to represent a mean domain scale score. An example of an item and response options, as well as clarification in italics, in the practical domain is:

Item nr 37: ‘Getting dressed and undressed’

1. Doesn't get dressed or undressed, not even with help.
2. Gets undressed and dressed with help.
3. Gets undressed or dressed on his/her own.

*May get help with buttons and closures*.

4 Gets undressed and dressed on his/her own

*May get help with buttons and closures*.

5 Gets undressed and dressed on his/her own, including all buttons and closures

*It is not necessary that the client be able to tie shoelaces*.

A mean total DIAB scale score is calculated by averaging the 67 mean domain scale scores for the three core adaptive behaviour domains (Conceptual, Practical, and Social). Higher DIAB total and mean domain scores represent better mastery of adaptive skills (i.e., higher adaptive behaviour). Scores closer to one represent complete or virtually complete absence of skills, which indicates a substantial need for support. Scores closer to five represent almost complete mastery of skills which indicates that less or little support with these skills is needed. Scores are given according to actual behaviour, not to what clients are potentially capable of. Hence, informants are instructed to choose per item at which level of mastery the client currently functions in most situations. Some items describe the level of mastery of a skill in terms of executing the skill with ascending levels of independence (less support is needed with every step towards mastery of the skill). For example: the skill on level 1 is executed with extensive support almost taking over the skill; the same skill on level 5 is executed by the client without any or very little support, depending on the complexity. Some skills do not lend themselves to include the level of support in the assessment of mastery of that skill. These skills are described in ascending levels of complexity without the level of support as a factor (for example: level 1 is having no language abilities, only producing sounds/idiosyncratic words; level 5 is using fluent speech).

#### 2.3.2 Adaptive Behaviour Assessed With the SRZ

The SRZ (Kraijer et al. 2004) is an adaptation of the ‘Cain‐Levine Social Competency scale’ (Cain et al. 1963). It has 31 items on adaptive behaviour aimed at adults (above 17 years of age) and children (above 4 years of age) with moderate and severe intellectual disability. It has norms for different age groups. Akin to Likert‐type scaling, items are represented in an ordered category format: each item has four options representing different well‐defined and ascending levels of mastery of an adaptive skill similar to the DIAB, resulting in raw scores from 1 (no mastery of the skill) to 4 per item (mastery of the skill appropriate for persons with a moderate intellectual disability). Four skill domains ‘self‐reliance’ (SR), ‘use of language’ (UL), ‘focus on tasks’ (FT) and ‘social orientation’ (SO) add up to a total scale score. Test properties of the SRZ were evaluated as adequate (Egberink and De Leng 2024; Kraijer 2000; Scheirs et al. 2012), based on an evaluation of test construction, quality of test material and manual, reliability, and criterion validity. SRZ raw total and domain scores were used in the current study. For the current study, UL and FT skill domains were combined because the content of the items in both skill domains fit the current domain of conceptual skills as described in DSM‐5‐TR (American Psychiatric Association 2022).

#### 2.3.3 Motor Skills Assessed With the SMZ

The SMZ (Kraijer and Kema 1994) is an instrument for gross motor skills developed for children (above 3 years of age) and adults with moderate and severe disabilities. It has norms for different age groups. The instrument consists of 22 dichotomous items to be scored with + (client performs the activity) or – (client does not perform the activity). A + score has a raw item score of 1, and a – score has a raw item score of 0. Items are ordered from low to high complexity of the skills. As for the SRZ, test properties were evaluated as adequate (Egberink and De Leng 2024; Kraijer 2000). SMZ raw total and domain scores were used in the current study.

#### 2.3.4 Global Rating of Adaptive Behaviour

The informants were asked to mark the overall adaptive behaviour skills on the following question: ‘Considering all clients with an intellectual disability you have known in your work as a care staff member, how would you grade the participant on adaptive behaviour (self‐reliance) from 1 (has no self‐reliance skills) to 10 (has much self‐reliance skills)’. The mark assigned by the informant to the participant was used as the global rating variable. In the Netherlands, the word ‘self‐reliance’ is commonly associated with support given in self‐care skills.

### 2.4 Statistical Analyses

The study protocol was pre‐registered on Open Science Framework (OSF; https://osf.io/sbj9e/?view_only=160d379d72e3487fbece46d01d1eb557; Anonymous 2022). The target sample size was set at 60, including three subgroups with each having 20 participants with moderate, severe, and profound intellectual disability, respectively. The target sample size was determined by conducting a power analysis with the software programme G*Power (Faul et al. 2007). We required a power of 0.90 for statistically testing an anticipated Pearson's *r* correlation coefficient of at least 0.60. For intraclass correlation coefficient (ICC) values a sample size with a total of 60 is sufficient to reach a 95% confidence interval of ±0.1 with ICC values of 0.80 or larger (two repeated measurements; De Vet et al. 2011).

IBM Statistical Package for Social Sciences (SPSS) version 28 was used. The five DIAB items with four scoring options (1–4) were recalibrated into a one‐to‐five‐point scoring scale. This resulted in: score 1 = 1, score 2 = 2.33, score 3 = 3.67, score 4 = 5.

Group differences in the moderate, severe and profound groups concerning demographic variables were assessed. A one‐way analysis of variance (ANOVA) test was conducted for age. Also, a *χ* ^2^ test was conducted to test for differences in gender. Results were used to assess whether the groups differed on characteristics other than severity of intellectual disability such as age or gender which may affect differences in mean scores between the three groups.

The Standard Error of Measurement, which here refers to the measurement error of a single client's total and domain DIAB scores, was calculated as a descriptive analysis using the square root of the error variance of the test–retest agreement according to Shrout and Fleis (1979) (also see De Vet et al. 2011). The error variance was derived from the variance components displayed in the output of the SPSS ANOVA table.

For the first research question, assessing known‐group validity, the mean total DIAB scores by Informant One of the three groups of clients with moderate, severe and profound intellectual disability were compared. First, differences between the three mean group scores were analyzed with a one‐way ANOVA test. Effect sizes were calculated as eta‐squared. Second, post hoc tests were conducted with ANOVA to test which severity groups had statistically different mean scores. Values of *p* smaller than 0.05 were considered statistically significant. Effect sizes were calculated as Cohen's *d* (mean difference divided by the pooled standard deviation per group dyad). Although the interpretation is field‐, content‐ and research method‐specific, empirically derived guidelines from social psychology were used (Lovakov and Agadullina 2021). Cohen's *d*s of 0.15, 0.36, and 0.65 were interpreted as small, medium, and large effects, respectively. Also, its magnitude was interpreted in the current context of the study. An effect size larger than 1 would be interpreted as having one skill level up or down, dependent on the severity of intellectual disability.

The second research question on convergent validity was answered using Pearson's *r* correlation coefficient between the total mean DIAB scale score of Informant Two (Measurement One) and the total SRZ scale score of Informant One. This was done for all correlation coefficients to prevent single informant bias. Also, the convergent validity of the Motor domain was assessed by calculating Pearson's *r* correlation coefficient between the mean Motor domain score and the total raw SMZ score. A minimum correlation coefficient of 0.60 was chosen as the benchmark for the convergent validity of a new instrument (Odom and Morrow 2006).

The third research question on concurrent validity was answered by calculating Pearson's *r* correlation coefficient between total DIAB scale scores and global ratings of adaptive behaviour by Informant One. Although global ratings were obtained from Informant One and Two, only the global rating of Informant One was used in the analysis because it had fewer missing values.

The fourth research question on assessing convergent validity and exploring discriminant validity of mean DIAB domain scores, we configured the reliability and validity coefficients according to a partial Multi Trait Multi Method (MTMM) matrix. A MTMM matrix (Campbell and Fiske 1959, as described by Kenny 2012 and Shen 2017) represents reliability measures (ICC measures), mono‐method validity and hetero‐method correlation coefficients (Pearson's *r* correlations) between total and domain scale scores of the DIAB, total and subscale scores of the SRZ and total scale score of the SMZ. In addition, correlation coefficients between all measures and global ratings were represented in the matrix, except for SRZ ratings of the different SRZ domains by the same respondent (only Informant 1 completed the SRZ).

For the fifth research question on interrater and test–retest reliability, ICC values for agreement (single measures) were calculated for the mean score of each of the four domains ‘Conceptual’, ‘Practical’, ‘Social’, and ‘Motor’ as well as the total mean DIAB scale score. We followed the recommendations of Koo and Li (2016) to use a one‐way random effects ANOVA for the interrater reliability and a two‐way mixed effects ANOVA to calculate ICC test–retest values. Test–retest ICC values were interpreted in accordance with the systematic review by Floyd et al. (2015) of studies on adaptive behaviour assessments, recommending ICC values with a minimum of 0.90 as the standard for total scale scores and 0.80 median for domain scores concerning adaptive behaviour instruments (as an amendment to the benchmarks in the pre‐registration). Floyd et al. (2015) employed a 0.60 standard for interrater reliability median for total composite and domain areas.

## 3 Results

### 3.1 Descriptive Analyses

Participant numbers varied across different measurements due to dropouts (see Figure 1). Attrition was not associated with client characteristics (i.e., age, gender, severity of intellectual disability). One DIAB assessment had missing values due to a technical problem. The global rating question was introduced when data collection was already underway. For this reason, we assumed these data were missing completely at random.

Regarding age, the one‐way ANOVA shows significant differences between the three groups moderate, severe, and profound intellectual disability regarding age (*F*(2, 67) = 3.33, *p* = 0.046). The post hoc Games‐Howell test shows non‐significant differences regarding age between the group moderate intellectual disability (*M* = 48.5, Mdn = 51.0, SD = 17.9) and the group severe intellectual disability (*M* = 38.6, Mdn = 37.5, SD = 17.1, *p* = 0.122), between the group moderate intellectual disability and the group profound intellectual disability (*M* = 37.2, Mdn = 33.0, SD = 13.0, *p* = 0.056), and also between the group severe intellectual disability and the group profound intellectual disability (*p* = 0.939). There were no significant differences between the three groups moderate, severe, and profound intellectual disability regarding gender (*χ* ^2^(1) = 3.37, *p* = 0.185). See Table 1 for counts on gender in the three severity groups.

In Table 2 all variance components, Standard Error of Measurement values and other measurement indices are displayed. For the mean total DIAB score the Standard Error of Measurement of the individual score, which runs from 1 to 5, had a value of 0.16. This means that in 95% of cases a person's total DIAB score can be expected to lie in the range between the observed score plus and minus 0.31 (1.96 times the Standard Error of Measurement). For the domain scores, Standard Error of Measurement values running from 0.19 to 0.27 were obtained.

**TABLE 2:** Standard error of measurement (SEM) of DIAB domains.

DIAB DOMAINS	*N*	*M* ^a^	SD^b^	Measurement variance^c^	Client variance^c^	Error variance^c^	SEM^e^
DIAB TOTAL	59	2.42	0.81	,00^d^	0.60	0.03	0.16
DIAB CON	59	2.32	0.88	,00^d^	0.67	0.04	0.19
DIAB PRAC	59	3.14	0.87	,00^d^	0.73	0.04	0.19
DIAB SOC	59	2.67	0.79	,00^d^	0.53	0.07	0.25
DIAB MOT	58	2.28	0.88	,00	0.72	0.07	0.27

*Note:* With the SEM the 95% confidence interval of the individual mean total or domain score can be calculated (mean score ±1.96 × SEM).

Abbreviations: *M* = mean score; *N* = number of informants; SD = standard deviation; SEM = standard error of measurement.

^a^ All scores are derived from mean total and domain scores.

^b^ SD of mean total and domain scores.

^c^ Derived from variance components.

^d^ SPSS sets negative variance values to 0 (no variance due to systematic differences between the two measurements, both by Informant Two). This was the case except for the motor domain.

^e^ Formula: √(Error Variance).

### 3.2 Known‐Group, Convergent, Discriminant and Concurrent validity

**FIGURE 2:**
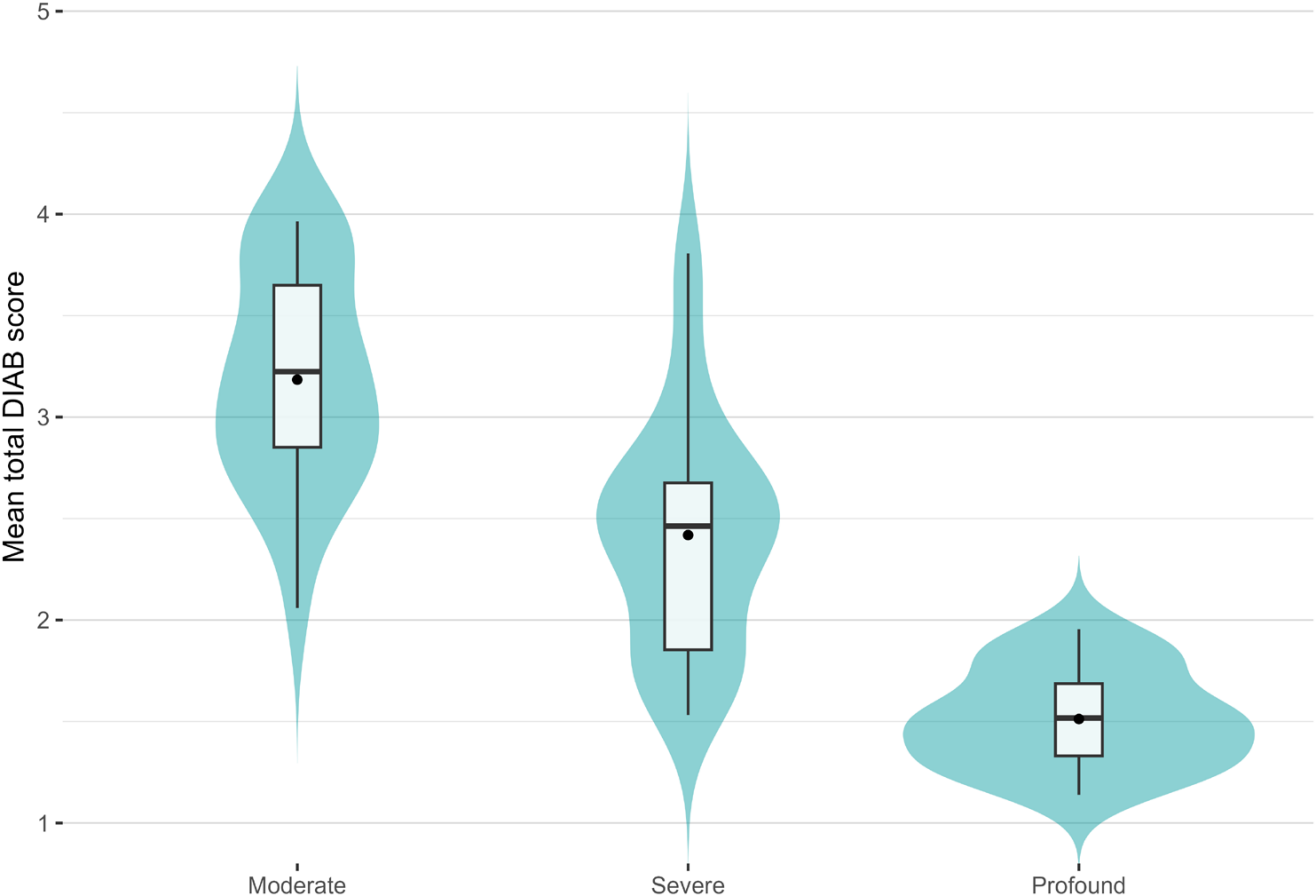
Distribution of mean total DIAB scores per subgroup. The mean total DIAB scores of the individuals of the three groups of participants separately: People with a moderate (*N* = 23), severe (*N* = 28) and profound intellectual disability (*N* = 19). Measured by Informant One. Black dot represent the mean. Differences between the groups ‘moderate Intellectual Disability‐severe Intellectual Disability’ and ‘severe Intellectual Disability‐profound Intellectual Disability’ were statistically significant at *p* < 0.001.

Regarding known‐group validity, differences in mean total DIAB scores between the three groups of clients with moderate, severe, and profound intellectual disability are shown in Figure 2 (black dotted lines are mean scores). The violin plot displays the expected density of DIAB mean total scores. Results of the one‐way ANOVA test show significant differences with a substantial effect between the three groups (*F*(2, 16.03) = 60.76, *η* ^2^ = 0.645, *p* < 0.001). *The post hoc Games‐Howell test showed significant* differences between all three dyads of the three groups with different severity levels (see Table 1 for mean and standard deviations per group), each difference between severity groups at *the p* < 0.001 level. Cohen's d effect sizes are 1.38 for the difference between the moderate and severe group, 2.08 for the difference between the severe and profound group, and 4.06 for the difference between the moderate and profound group, all large effects. In practise, it would mean that on average clients with a moderate intellectual disability performed DIAB skills more than one skill level higher than clients from the severe intellectual disability *group*; for example: from level 3, performing the skill with some help, to level 4, performing the skill with almost no help. Also, clients with severe intellectual disability performed skills on average more or less two skill levels higher than clients from the profound group. Last, clients from the moderate group performed skills on average more or less four skill levels higher than *clients* from the severe group.

Convergent validity was indicated by the statistically significant and very strong correlation coefficient between total mean DIAB scale score and total SRZ scale score (*r* = 0.90; *p* < 0.001, *N* = 67; Table 3). This also applied to the statistically significant and strong correlation coefficient between the mean Motor domain score and the total raw SMZ score (*r* = 0.77; *p* < 0.001, *N* = 66; Table 3).

**TABLE 3:** Partial multi trait multi method table.

	Method 1: DIAB^a^ informant two				
	DIAB TOTAL^c^	DIAB CON	DIAB PRAC	DIAB SOC	DIAB MOT
Method 1: DIAB^a^ informant one					
DIAB TOTAL^c^	0.94***	0.91***	0.91***	0.90***	0.71***
DIAB CON	0.92***	0.91***	0.86***	0.88***	0.66***
DIAB PRAC	0.92***	0.86***	0.93***	0.83***	0.76***
DIAB SOC	0.86***	0.84***	0.79***	0.87***	0.58***
DIAB MOT	0.78***	0.73***	0.82***	0.68***	0.84***
Method 2: SRZ/SMZ^b^ informant one					
SRZ TOTAL	0.90***	−	−	−	0.74***
SRZ UL + FT	−	0.93***	0.80***	0.83***	0.61***
SRZ SR	−	0.75***	0.87***	0.70***	0.81***
SRZ SO	−	0.70***	0.69***	0.77***	0.47***
SMZ	0.48***	0.40***	0.58***	0.38***	0.77***
Method 3: Global rating informant one					
Global rating	0.56***	0.51***	0.57***	0.54***	0.58***

*Note:* The upper diagonal of the correlation matrix contains ICC values. The matrix is not symmetric because reliability is not being measured for methods 2. and 3. Correlation coefficients were not relevant for SRZ domain scores and DIAB domain scores with the total SRZ score because domain scores share variance with total scores of the same instrument.

*** *p* < 0.001.

^a^ All DIAB scores are mean total and mean domain scores.

^b^ All SRZ/SMZ scores are total scores. SRZ and SMZ were both used as ‘Method 2’ because we considered SMZ as a single skill domain (motor skills).

^c^ DIAB TOTAL is the mean of the domains DIAB CON, DIAB PRAC and DIAB SOC.

Concerning concurrent validity, associations between global ratings of adaptive behaviour and DIAB total and domain scores were statistically significant and moderate with values between 0.51 and 0.58 (*p* < 0.001, *N* = 41). The highest values were found for the associations between the global rating and motor skills (0.58), and between the global rating and practical skills (0.57).

Table 3 displays the convergent and discriminant validity indices within a partial MTMM matrix. In general, all correlation coefficients were statistically significant and predominantly moderate to very strong, including the associations between conceptually distinct sub‐constructs, such as the Social domain (SO) of the SRZ and the Conceptual domain (CON) of the DIAB. One exception was the weak association between DIAB social skills and SMZ motor skills (*r =* 0.38). More specifically, the association between the domains of the DIAB and similar domains of the SRZ/SMZ (same traits–different methods; i.e., the DIAB and the SRZ/SMZ) was all statistically significant, positive, and strong to very strong. This provided support for the convergent validity of the DIAB domains, in addition to the previously described very strong correlation coefficient between total DIAB and total SRZ scores. Regarding the discriminant validity of the domain scores, associations between different domains of the DIAB and those of the SRZ/SMZ (different traits–different methods) ranged from 0.38 to 0.83 (weak to strong): four out of nine correlation coefficients were as expected. From the four correlation coefficients that were as expected (moderate or weak), three were associations between DIAB domains and SMZ motor skills (0.38 to 0.58). Associations between different domains within the DIAB (different traits–same method) ranged from 0.58 to 0.88. Although no strict benchmarks exist for discriminant validity and values depend on how close constructs are related (Rönkkö and Cho 2022), the moderate to strong between‐domain associations may indicate limited discrimination between the domains of the DIAB.

#### 3.2.1 Inter‐Rater and Test–Retest Reliability

Table 4 contains the correlation coefficients between DIAB mean total and domain scores as well as between different raters and rating moments. Correlation coefficients within one measurement (by one informant) and between different DIAB domain scores (except for the motor domain) were all statistically significant and strong to very strong. The motor domain showed moderate to strong associations with the other DIAB domains. Inter‐rater reliability of the total mean DIAB score (ICC = 0.94, *N* = 66; Table 4) met test standards for adaptive behaviour instruments. This was also the case for mean DIAB domain scores (all four domains: Mdn = 0.89, *N* = 66; three core domains: Mdn = 0.91, *N* = 66). Test–retest reliability of the DIAB as completed by the same informant was somewhat higher than interrater reliability. The ICC of the total mean DIAB score amply met the 0.60 benchmark (total mean DIAB score: ICC = 0.96, *N* = 59; all four domains: Mdn = 0.93, *N* = 59; three core domains: Mdn = 0.95, *N* = 59; Table 4 *)*.

**TABLE 4:** Correlation matrix of DIAB scores for different informants and measurements.

	DIAB^a^ informant one^b^					DIAB^a^ informant two M1^c^					DIAB^a^ informant two M2^d^				
	DIAB TOT	DIAB CON	DIAB PRAC	DIAB SOC	DIAB MOT	DIAB TOT	DIAB CON	DIAB PRAC	DIAB SOC	DIAB MOT	DIAB TOT	DIAB CON	DIAB PRAC	DIAB SOC	DIAB MOT
DIAB^a^ informant one^b^															
DIAB TOT	1														
DIAB CON	0.97***	1													
DIAB PRAC	0.96***	0.88***	1												
DIAB SOC	0.94***	0.88***	0.83***	1											
DIAB MOT	0.78***	0.73***	0.84***	0.63***	1										

	TOT	CON	PRAC	SOC	MOT	TOT	CON	PRAC	SOC	MOT	TOT	CON	PRAC	SOC	MOT
DIAB^a^ informant two M1^c^															
DIAB TOT	0.94***	−	−	−	−	1									
DIAB CON	−	0.91***	0.86***	0.84***	0.73***	0.97***	1								
DIAB PRAC	−	0.86***	0.93***	0.78***	0.82***	0.96***	0.89***	1							
DIAB SOC	−	0.89***	0.83***	0.87***	0.67***	0.94***	0.92***	0.83***	1						
DIAB MOT	−	0.66***	0.76***	0.58***	0.84***	0.73***	0.68***	0.81***	0.57***	1					

	TOT	CON	PRAC	SOC	MOT	TOT	CON	PRAC	SOC	MOT	TOT	CON	PRAC	SOC	MOT
DIAB^a^ informant two M2^d^															
DIAB TOT	0.92***	−	−	−	−	0.96***	0.94***	0.91***	0.91***	0.69***	1				
DIAB CON	−	0.88***	0.83***	0.78***	0.68***	0.93***	0.95***	0.84***	0.89***	0.62***	0.96***	1			
DIAB PRAC	−	0.85***	0.93***	0.74***	0.82***	0.92***	0.86***	0.95***	0.80***	0.79***	0.97***	0.86***	1		
DIAB SOC	−	0.82***	0.74***	0.76***	0.55***	0.87***	0.87***	0.77***	0.90***	0.50***	0.93***	0.87***	0.80***	1	
DIAB MOT	−	0.66***	0.77***	0.52***	0.86***	0.71***	0.64***	0.78***	0.58***	0.90***	0.75***	0.65***	0.85***	0.60***	1

*Note:* The diagonal of the block of correlation coefficients between Informant One and Informant Two First measurement (interrater reliability), and between Informant Two First measurement and informant two second measurement (test–retest reliability) contains ICC values.

^a^ All DIAB scores are mean total and mean domain scores.

^b^ There was only one measurement by informant one (in the same week as the first measurement of informant two).

^c^ M1 = First measurement (DIAB filled in) by Informant Two.

^d^ M2 = Second measurement by Informant Two (3 weeks after M1).

*** *p* < 0.001.

## 4 Discussion

This study tested the psychometric properties of the DIAB, a new instrument for assessing adaptive skills in people with moderate to profound intellectual disability. We found strong support for convergent, concurrent, and known‐groups validity. We found additional support for the convergent validity of the DIAB domains in the partial MTMM matrix: associations between the same domains of the DIAB and the SRZ/SMZ were strong to very strong. Concerning discriminant validity, the pattern of correlations as displayed in the partial MTMM matrix was partly congruent with expectations. For conceptually the most distinct constructs (motor skills measured by SMZ and DIAB core domains conceptual, practical and social skills) the moderate to weak associations were in line with expectations. However, associations between DIAB core domains and different subconstructs measured by SRZ were mainly strong. Interrater and test–retest reliability for total and domain scores were in accordance with standards set by Floyd et al. (2015). Reliability values were also comparable to those in more recent studies on other adaptive behaviour instruments, which indicate test–retest and interrater reliability for ABAS‐3 ranging from 0.70 to 0.90 (Harrison and Oakland 2018; Pepperdine and McCrimmon 2017) and for Vineland‐3 ranging from 0.61 to 0.92 (Von Buttlar et al. 2021). The DIAB test–retest and interrater reliability values ranged from 0.84 to 0.96.

The results of this first study of the DIAB, aimed at overcoming floor effects of existing instruments, are encouraging, on the basis of which suggestions for future research can be given. Correlation coefficients between the different DIAB domains for the same rater in one measurement were strong to very strong. Although the DIAB domains are based on several factor analytic studies on which the current diagnostic manuals are based (American Psychiatric Association 2022; Tassé et al. 2012; WHO 2022), these factor analytic studies were conducted in broader populations. Adaptive behaviour domains may be different for people with moderate to profound intellectual disability than for people with mild intellectual disability, as De Bildt et al. (2005) found in children and adolescents with the Dutch version of the Vineland Adaptive Behaviour Scales (VABS), especially for the severe and profound group. Research is therefore needed on the factorial structure of adaptive behaviour domains in groups with moderate to profound intellectual disability and how these domains may be reflected in the DIAB. In light of the strong associations amongst domains, sample size needs to be large for testing factor structure as well as for testing the pattern of correlations in a MTMM matrix.

A limitation of the study is that the measures used to explore convergent validity are only used in the Netherlands. Although these instruments have good measurement properties (Egberink and De Leng 2024; Kraijer 2000), they were validated in the 1970s and 1980s, when adaptive behaviour was conceptualised differently (Tassé et al. 2012). If the DIAB is also to be used in other countries, it would not only require a translation into other languages and validation to other cultural contexts, but also establishment of validity in relation to local diagnostic assessment practises for adaptive behaviour in people with moderate to profound intellectual disability. Furthermore, caution is needed with respect to the generalizability of the sample, since information on aetiology and ethnicity was not collected in the current study. Another limitation is the lack of information regarding the length of time informants have worked with the clients about whom they completed the DIAB, except for the required minimum of 3 months in this study.

With future research on the DIAB with larger sample sizes we can replicate and refine the current distribution of scores and mean scores of the DIAB in people with moderate, severe and profound intellectual disability. Additionally, research should be done on the sensitivity to change of DIAB scores, associations between DIAB scores and intensity of care support, DSM‐5(TR) and medical diagnoses to further confirm discriminative validity and to test hypotheses on associations between DIAB scores and these variables. For example, we expect a negative association between support intensity and adaptive behaviour skills. Finally, discriminant validity needs to be further tested by comparing DIAB scores against constructs such as challenging behaviours (e.g., self‐injury, aggressive behaviour) and mental health problems.

In sum, this study shows support for the validity and reliability of the DIAB. Its outcomes justify further research on the DIAB with larger samples. This may lead to a scientifically validated instrument, which can help diagnosticians classify severity levels and plan support for people with moderate, severe or profound intellectual disability. Having a valid and reliable instrument to distinguish between moderate to profound intellectual disability will help with matching care intensity and type to needs, monitoring whether care that is provided is still adequate, and with building arguments for obtaining the level of funding needed to deliver such care. If the structure of the DIAB holds up in further testing, it may support differentiated planning and evaluations of interventions on adaptive behaviour.

## Conflicts of Interest

Hinke Elisabet Drijver is the author of the DIAB as an employee of care organization ‘s Heeren Loo. In that capacity, development and initial testing were funded by charity organisations.

## Supporting information


**Data S1:** Supporting Information.

## Data Availability

The data that support the findings of this study are available on request from the corresponding author. The data are not publicly available due to privacy or ethical restrictions.

## Endnotes

^1^
In the context of this study and the Dutch care system for people with intellectual disability, diagnostic assessments of behaviour and functioning are done by psychologists or ‘orthopedagogen’. ‘Orthopedagoog’ is a Dutch term referring to an academically trained, licenced special educator offering guidance and treatment in deviant, problematic parenting and development situations, for example, in case of a developmental or learning delay or an impairment.
