# Supplementary material for: Reliability and Validity of the Diagnostic Instrument on Adaptive Behaviour: A New Instrument Measuring Adaptive Behaviour in People With Moderate, Severe or Profound Intellectual Disability
Source: J Appl Res Intellect Disabil. 2025 Nov 14;38(6):e70150. doi: 10.1111/jar.70150 (PMC12618112; doi:10.1111/jar.70150)
Supplement: Supplementary file 1 — Data S1: Supporting Information. [file JAR-38-e70150-s001.docx]

**Supplement: the development of the Diagnostic Instrument on Adaptive Behavior (DIAB)**

The development and construction of the DIAB were conducted from October 2019 to November 2021. In the diagnostic assessment of people with a moderate or severe intellectual disability, the SRZ (‘Sociale Redzaamheidsschaal-Z’, English: social competency scale for people with intellectual disability; Kraijer et al., 2004) to measure adaptive behavior skills was (and still is) common practice in the Netherlands, and modern alternatives were hardly available for these people. The SRZ is used in the diagnostic assessment of people with moderate or severe intellectual disability. Despite extensive use of the SRZ especially at the lower range of skill mastery the instrument became outdated and does not capture the full range of skills of people with profound intellectual disability anymore. The objective of the steps taken to develop the Diagnostic Instrument Adaptive Behavior (DIAB), was to develop an up-to-date alternative to the SRZ with items specifically suitable for people with moderate to profound intellectual disability. The aims were: (1) developing a test structure in accordance with most current standards, with items suitable for people with moderate to profound intellectual disability, (2) involving professionals, clinicians and direct care staff members supporting these people in the process of the development of the DIAB, and (3) pilot testing the DIAB with help of these professionals.

**Steps followed to develop items**

The DIAB was developed following six steps. First, from October 2019 to March 2020 a test structure was defined according to the most current definition and international standards of adaptive behavior as described in DSM-5, ICD-10 and AAIDD handbook (American Psychiatric Association, 2013; Schalock et al., 2010; Tassé et al., 2012; World Health Organization, 1993).

Second, from March to July 2020, items were constructed according to skills appropriate for people with moderate to profound intellectual disability, hence in the low range of the distribution in the general population. The SRZ only had items aimed at the moderate and severe range and the ABAS-3 (Adaptive Behavior Assessement System; Harisson & Oakland, 2015, 2020) and Vineland-II (Sparrow, Cicchetti & Balla, 2005) only had items aimed at the general population and people with a mild intellectual disability.

The researcher’s own experience in the field (she is a consulting diagnostician with extensive experience working with people with moderate to profound intellectual disability) was applied next to the consultation of a group of 19 professionals (16 on Master's level, three on Bachelor's level). These professionals worked in 11 residential care facilities for and with people with intellectual disability. Professionals were consulted in five survey rounds using an online questionnaire format. They were asked which skills an adaptive behavior instrument should consist of, how different clearly described levels of skill mastery could be distinguished, and which skills were missing in the instruments used at that moment (an example of a modern skill was ‘handling a tablet or mobile phone’).

Third, from July to October 2020, results of the questionnaires were processed and a first complete draft of the DIAB was constructed with help of a five-step task analysis. Five items resulted in four steps of ascending levels of one skill.

Fourth, in October 2020 feedback on the draft of the DIAB was collected in two rounds. In round one, feedback concerning face validity and content was collected from diagnosticians who used instruments on adaptive behavior (e.g. SRZ or other, less suitable instruments) on a regular basis, working in residential care facilities for people with intellectual disability. They were asked to read the complete DIAB thoroughly and whether it measured adaptive behavior comprehensively and/or if they missed items. They were also asked to give suggestions for improving items concerning their content and comprehensibility. In round two, several direct care staff members were asked to review the DIAB on comprehensibility as a test takers’ pilot. Their feedback was processed and the DIAB was adapted accordingly.

Fifth, from July to November 2021, a scaling analysis was conducted. Five ascending levels of each skill of the DIAB were randomly rearranged per item and presented to 17 professionals working in residential care facilities for and with people with intellectual disability. The aim was to investigate if the sequence of the five ascending levels per skill made sense and was correct. The professionals were asked to put the five levels of each skill in the right order (five items had four levels), from least skillful to most skillful per item. Items which were not correctly ordered according to the constructed DIAB by less than 75% of the participants were inspected and adapted. The final draft of the DIAB was the basis for the current study to test validity and reliability.

**References**

American Psychiatric Association. (2013). *Diagnostic and statistical manual of mental disorders (5th ed.)*. American Psychiatric Association Publishing.

Harrison, P. L. & Oakland, T. (2015). *Adaptive Behavior Assessment System (3rd ed.)* [Manual]. Western Psychological Services.

Harrison, P. L. & Oakland, T. (2020). *ABAS-3 Schaal voor adaptief gedrag. Instructies en verantwoording*. Hogrefe Uitgevers B.V.

Kraijer, D. W., Kema, G. N., & de Bildt, A. A. (2004). *SRZ/SRZ-i. Sociale Redzaamheids-*

*Schalen [Social Competency Scale for People with Intellectual Disabilities] [Manual]*. Pearson Benelux B.V.

Schalock, R. L., Borthwick-Duffy, S. A., Bradley, V. J., Buntinx, W. H. E., Coulter, D. L., Craig, E. M., Gomez, S. C., Lachapelle, Y., Luckasson, R., Reeve, A., Shogren, K. A., Snell, M. E., Spreat, S., Tasse, M. J., Thompson, J. R., Verdugo-Alonso, M. A., Wehmeyer, M. L., & Yeager, M. H (2010). *Intellectual disability: Definition,*  d*iagnosis, classification, and systems of support* (11th ed.). American Association on Intellectual and Developmental Disabilities.

Sparrow, S. S., Cicchetti, D. V., & Balla, D. A. (2005). Vineland Adaptive Behavior Scales (2nd ed.). American Guidance Service.

Tassé, M. J., Schalock, R. L., Balboni, G., Bersani, H. Jr., Borthwick-Duffy, S. A., Spreat, S., Thissen, D., Widaman, K. F., & Zhang, D. (2012). The construct of adaptive behavior: its conceptualization, measurement, and use in the field of intellectual disability. *American Journal on Intellectual and Developmental Disabilities*, *117*(4), 291-303. https://doi.org/10.1352/1944-7558-117.4.291

World Health Organization (1993). *The ICD-10 classification of mental and behavioural*  *disorders: diagnostic criteria for research.* World Health Organization.
